# Self-Generated Lower Body Negative Pressure Exercise: A Low Power Countermeasure for Acute Space Missions

**DOI:** 10.3390/life14070793

**Published:** 2024-06-24

**Authors:** Suhas Rao Velichala, Ryan D. Kassel, Victoria Ly, Donald E. Watenpaugh, Stuart M. C. Lee, Brandon R. Macias, Alan R. Hargens

**Affiliations:** 1 Department of Orthopaedic Surgery, UC San Diego Medical Center, University of California San Diego, La Jolla, CA 92093, USA; ryan.kassel@colorado.edu (R.D.K.); vily@ucsd.edu (V.L.); ahargens44@gmail.com (A.R.H.); 2 Department of Physiology and Anatomy, University of North Texas Health Science Center, Fort Worth, TX 76107, USA; 3 National Aeronautics and Space Administration, Huntsville, AL 35808, USA; stuart.lee-1@nasa.gov (S.M.C.L.); brandon.r.macias@nasa.gov (B.R.M.)

**Keywords:** spaceflight, microgravity, SANS, LBNP, fluid shifts

## Abstract

Microgravity in spaceflight produces headward fluid shifts which probably contribute to Spaceflight-Associated Neuro-Ocular Syndrome (SANS). Developing new methods to mitigate these shifts is crucial for preventing SANS. One possible strategy is the use of self-generated lower body negative pressure (LBNP). This study evaluates biological or physiological effects induced by bed rest to simulate adaptations to microgravity. Participants were tested during powered LBNP and dynamic self-generated (SELF) LBNP at 25 mmHg for 15 min. The results were compared to the physiologic responses observed in seated upright and supine positions without LBNP, which served as controls for normal gravitational effects on fluid dynamics. Eleven participants’ (five male, six female) heart rates, blood pressures, and cross-sectional areas (CSA) of left and right internal jugular veins (IJV) were monitored. Self-generated LBNP, which requires mild to moderate physical activity, significantly elevated heart rate and blood pressure (*p* < 0.01). Self-generated LBNP also significantly reduced right IJV CSA compared to supine position (*p* = 0.005), though changes on the left side were not significant (*p* = 0.365). While the effects of SELF and traditional LBNP on IJV CSA were largely similar, traditional LBNP significantly reduced IJV CSA on both sides. Given its low mass, volume, and power requirements, SELF LBNP is a promising countermeasure against SANS. Results from this study warrant longer-term studies of SELF LBNP under simulated spaceflight conditions.

## 1. Introduction

On Earth, postural changes generate hydrostatic forces which alter intracranial pressure (ICP). In microgravity, astronauts experience a headward fluid shift because there is no gravitational vector to produce a hydrostatic gradient [1]. This leads to facial edema and a mild but chronically elevated ICP, which generates a chronic, mildly elevated gradient between ICP and intraocular pressure (IOP) [2]. This translaminar pressure gradient may at least partially underlie the pathogenesis of Spaceflight-Associated Neuro-Ocular Syndrome (SANS) [3]. 

In 2011, Mader and associates published a landmark paper that identified ocular pathologies in astronauts after long-duration spaceflight [4]. They hypothesized that cephalad fluid shifts and venous congestion may cause ocular structural and functional changes. The cephalad fluid shifts specifically impact volume and outflow in the internal jugular vein (IJV) which is responsible for venous outflow from the head [5]. Internal jugular vein cross sectional area (CSA) is related to ICP; it has been hypothesized that elevated venous pressure, indicated by increased IJV CSA, may impede cerebrospinal fluid absorption leading to increased ICP [6]. Therefore, we expect trends in IJV CSA to reflect trends in ICP. 

Lower body negative pressure (LBNP) offers a potential countermeasure to reverse cephalad fluid shifts in microgravity. Researchers explored this concept and found that traditionally powered LBNP effectively reduced ICP, IOP, and IJV CSA in simulated microgravity [3,7,8,9,10,11,12,13]. More recent studies indicate that daily LBNP exposure reduces choroidal thickening observed in head down tilt [13]. Several publications have also demonstrated that LBNP in the supine position, which simulated microgravity, decreases ICP, but not to upright levels [3,7,10,12,13,14].

Marshall-Goebel et al. demonstrated that LBNP reduces IJV CSA and improves IJV flow patterns in astronauts in microgravity [14]. Ground-based tests indicate that LBNP is effective for reversing fluid shifts [12] and LBNP may help reduce choroidal engorgement [13]. However, recent spaceflight data indicate that LBNP may not reduce choroidal engorgement in microgravity [9]. Although the physiological responses and efficacy are not completely established, LBNP remains a promising countermeasure against headward fluid shifts in microgravity. 

Traditional LBNP chambers are large, immobile, heavy, and electrically powered. Volume, mass, and power are limited resources in spaceflight. A low mass, collapsible, low-power LBNP device would minimize the impact of LBNP on spacecraft resources, especially for deep-space missions [15]. The purpose of this study is to evaluate a self-powered LBNP device and compare acute physiological responses between a traditional LBNP chamber and a self-generated LBNP chamber. We hypothesize that self-generated LBNP will produce similar physiological responses when compared to a traditional LBNP chamber in a model of simulated microgravity. 

## 2. Materials and Methods

### 2.1. Participants

Eleven healthy volunteers participated in the experiment. There were 6 males and 5 females; mean (sd) weight: 65.2 (15.0) kg; height: 170.6 (10.1) cm; mean waist circumference: 79.4 (11.0) cm. A power analysis was conducted to determine that the sample size was sufficient to establish significance for the study. As a group, subjects were of average fitness based on their height and weight according to the center for disease control (CDC). According to the CDC, average fitness is typically assessed using Body Mass Index (BMI) and physical activity levels. For adults, a BMI between 18.5 and 24.9 is considered normal, which aligns with our participants’ BMI range. Additionally, fitness levels were corroborated by self-reported physical activity levels, ensuring participants met the CDC’s recommendations of at least 150 min of moderate-intensity aerobic activity per week. Participants were selected for the trial based on height between 155 and 193 cm and a BMI of less than 30 kg/m^2^ to represent the prototypical astronaut size. Subjects were excluded if their waist size prevented them from comfortably sliding into the chamber or if there was too much space in between the subjects’ waist and chamber seal. Subjects gave informed voluntary consent before participating in the study.

### 2.2. Device Description

#### 2.2.1. SELF Device

The self-generating lower body negative pressure (SELF) device is a longitudinally collapsible but axially stable cylindrical chamber that encloses the user’s abdomen and legs. Each end of the cylindrical chamber consists of a rigid circular plate. An elliptical opening with a neoprene skirt and belt provides a waist seal. Metal rings maintain the structure of the chamber, allowing it to expand and contract without changing its diameter. The SELF device is similar to the device developed and tested by Watenpaugh et al. in 1999, except the SELF device in this study has manually operated valves instead of a passive one-way flap that allows air to exit the chamber during cylinder contraction [15]. 

**Figure 1:**
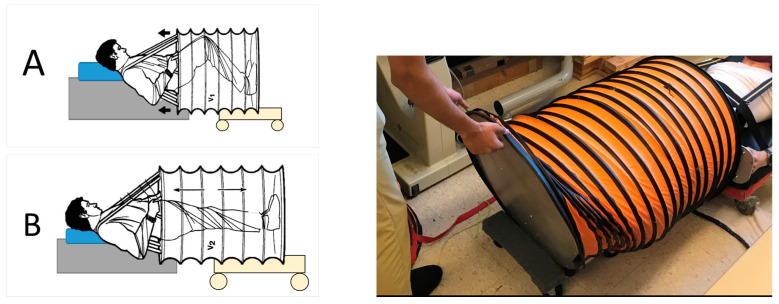
SELF LBNP. (**A**). With the valves open, a user contracts their legs to contract the SELF device. (**B**). A user then closes the valves and extends their legs. Expansion of the sealed cylinder generates negative pressure within the device. Arrows represent in which way the chamber extends. V1/V2 represent the two different positions in which the chamber may exist.

During SELF LBNP, a raised platform supports the upper body, and a moveable platform positioned beneath the bottom metal plate of the chamber allows the device to expand and collapse while in a horizontal position (Figure 1). The user wears a vest attached to the top plate of the chamber which distribute loads evenly across the shoulders. Handles on the top metal plate improve stability during use. 

#### 2.2.2. Traditional LBNP Chamber

**Figure 2:**
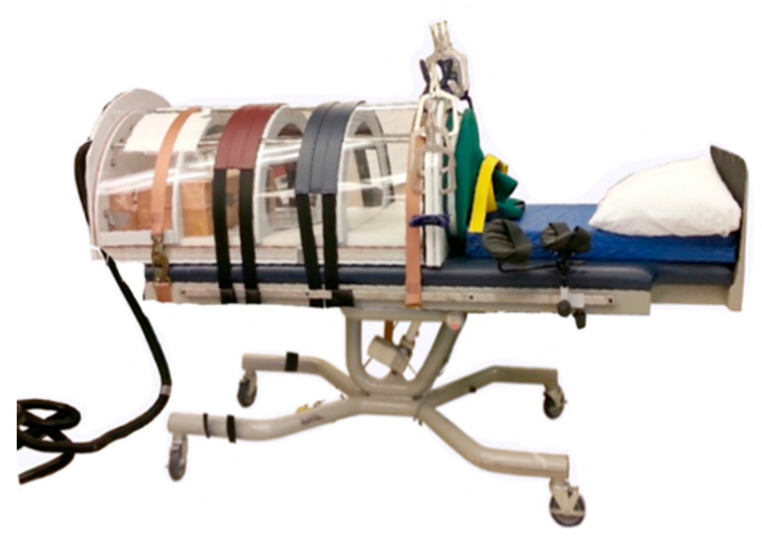
The traditional LBNP chamber. A powered vacuum attaches to the chamber to reduce internal pressure. The chamber is positioned horizontally with the user in supine posture.

The traditional LBNP chamber is a rigid, hemicylindrical chamber that encloses the lower body (Figure 2). A neoprene skirt, belt, and shoulder straps maintain a seal around the waist. A vacuum attached to the chamber generates negative pressure. 

#### 2.2.3. Protocol

Participants were evaluated under four conditions: seated upright posture, supine posture, traditional lower body negative pressure (LBNP), and self-generated LBNP. Heart rate, blood pressure, and cross-sectional areas (CSA) of the internal jugular veins (IJV) were the outcome measures assessed in this study. LBNP trials were 15 min each and were carried out at 25 mmHg in supine posture. Supine posture served as an analog for microgravity [16]. To ensure accurate and consistent measurements, participants first spent 10 min in seated upright or supine posture to adapt to each trial’s respective posture. The order of conditions was semi-randomly assigned for each subject such that two LBNP conditions were not consecutive. All trials were completed during a 1.5 h, single day study. Regarding the duration of treatment, while our study indicates that 1.5 h is sufficient to observe acute physiological changes, it is essential to clarify that this timeframe is focused on short-term effects. Future studies should explore longer durations to fully understand the chronic adaptations to microgravity.

During the supine, upright and traditional LBNP conditions, subjects remained stationary at rest. In the SELF LBNP condition, subjects performed repetitive leg press movements to generate negative pressure. Subjects were instructed to maintain 25 mmHg for as long as possible by extending their legs with the valves closed. Small leaks in the SELF device eventually caused the chamber to lose pressure. Once pressure fell to approximately 22 mmHg, subjects opened the adjustable valves of the chamber to equilibrate pressure inside the device. Subjects returned both legs into a flexed position, closed the valves, and extended their legs to begin the dynamic process again. This procedure was repeated for the duration of the trial. Subjects practiced these dynamic motions before the trial to ensure they could consistently reach 25 mmHg.

### 2.3. Data Acquisition and Analysis

Cross-sectional images of both left and right IJV were obtained via ultrasonography (Phillips, Amsterdam, The Netherlands). Images were taken in triplicate, just caudal to the bifurcation of the common carotid artery. IJV CSA was measured using image computing software (3D Slicer) (version 5.6.2) by two independent sonographers. For each set of images, sonographers identified and analyzed the smallest IJV CSA just before a carotid pulse. The Finometer system (Finapres, Enschede, The Netherlands) monitored and recorded subjects’ heart rate and blood pressure continuously throughout the experiment.

### 2.4. Statistical Analysis

A MANOVA was used to determine if there were significant differences between time points within a single LBNP trial. This acute study was further analyzed using a non-parametric Friedman’s Test. Wilcoxon signed-rank tests compared conditions. Bonferroni corrections were applied to address multiple comparisons. *p*-values less than 0.05 were considered significant.

A priori power analysis was conducted using G*Power software (version 3.1.9.7) to determine the appropriate sample size for detecting significant differences in internal jugular vein (IJV) cross-sectional area (CSA), heart rate, and blood pressure across conditions. The analysis indicated that a sample size of 11 participants would provide a power of 0.80 to detect a medium effect size (f = 0.25) with an alpha level of 0.05 for repeated measures ANOVA.

Effect sizes were calculated using Cohen’s d for pairwise comparisons. The effect size for the reduction in right IJV CSA during SELF LBNP compared to the supine condition was 0.76, indicating a medium to large effect. For the heart rate and blood pressure measurements, the effect sizes were 1.2 and 0.85, respectively, indicating large effects.

These effect sizes highlight the robustness of the findings despite the small sample size. Including these measures of effect size enhances the validity and interpretability of our results, addressing the reviewer’s concerns about the adequacy of the sample size and the strength of the observed effects.

## 3. Results

The experimental protocol was well tolerated by all subjects and no adverse events occurred. All subjects properly operated the device after a brief training period. 

Operation of the self-generating lower body negative pressure (SELF LBNP) device required mild to moderate exertion, and as expected, heart rate and blood pressure were both significantly higher compared to resting conditions (*p* < 0.01 for both). There were no other significant differences in heart rate or blood pressure. We observed large variances in both heart rate and blood pressure during SELF LBNP trials. We believe operation of the valves and gripping of the support handles interfered with the finger-based cardiovascular measurements leading to unreliable or missing data. Despite the limitations, the data still indicated significance. The amount of missing data did not affect the results.

We utilized a MANOVA statistical test to determine if internal jugular vein (IJV) cross sectional area (CSA) measurements varied over time. There were no significant differences between time points (left *p* = 0.773, right *p* = 0.161), indicating that IJV CSA did not significantly differ over the course of 15 min of traditional or SELF LBNP. Because IJV CSA did not vary with time, the three time point measurements were averaged. We did not observe significant differences between male and female subjects. There were significant differences between the left and right IJV CSA, so each side was analyzed separately. 

A Friedman’s test indicated that there were significant differences between conditions (*p* < 0.0005). Wilcoxon signed-rank tests evaluated pairwise comparisons between upright, supine, SELF LBNP, and traditional LBNP with Bonferroni corrections for multiple comparisons (Table 1). 

SELF LBNP was not significantly different when compared to traditional LBNP (left *p* = 0.465, right *p* = 0.577). However, traditional LBNP significantly reduced IJV CSA on both sides (left IJV *p* = 0.001, right IJV *p* = 0.005). A significant reduction on both sides suggests traditional LBNP may be more effective at reducing IJV CSA.

**Figure 3:**
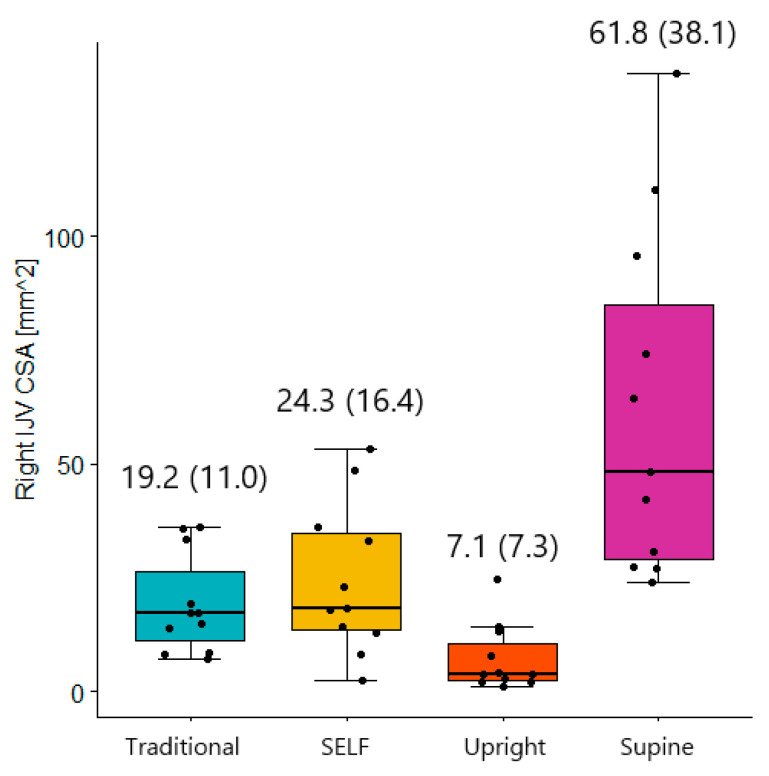
Right IJV CSA across different conditions. Mean (standard deviation).

**Figure 4:**
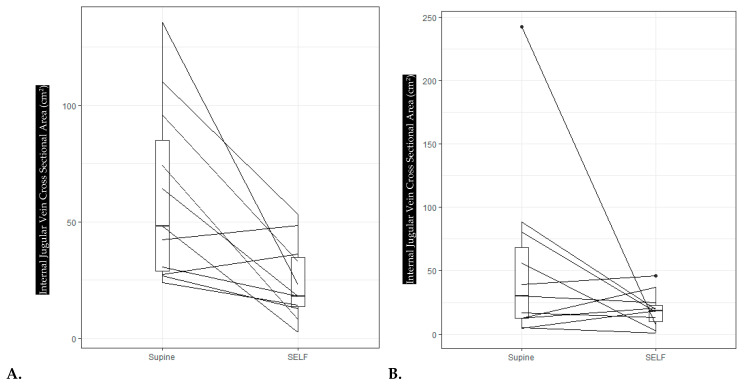
(**A**). Paired box plot of supine to SELF on Right IJV CSA. Note only 2 subjects increased IJV CSA. (**B**). Paired box plot of supine to SELF on Left IJV CSA. Note a much less pronounced downward trend.

Right IJV CSA during SELF LBNP was significantly smaller when compared to supine posture (*p* = 0.005, Figure 3). Figure 4A is a paired box plot showing differences in right IJV CSA between supine and SELF LBNP. SELF LBNP was significantly different when compared upright posture as well (*p* = 0.002). These results indicate SELF LBNP reduced right IJV CSA when compared to supine, but not to upright levels. 

Left IJV CSA was significantly reduced with traditional LBNP (*p* = 0.001), but not SELF LBNP (*p* = 0.365), when compared to supine posture. However, left IJV CSA supine values were significantly different from upright (*p* = 0.032). Figure 4B is a paired box plot that compares left IJV CSA between supine and SELF LBNP. 

## 4. Discussion

Our data support our hypothesis, suggesting that both traditional and SELF LBNP have similar effects on IJV CSA. It is important to note that these effects are induced by bed rest, which simulates adaptations to microgravity. SELF LBNP requires exercise to operate the device, and as expected, both heart rate and blood pressure were significantly higher when compared to traditional LBNP. 

We found both traditional and SELF LBNP significantly reduced right IJV CSA, but not to upright levels. These results are in accordance with several previous studies that found that LBNP reduces IJV CSA (or other related measures), but not to upright levels [3,7,10,12,13,14].

Our results have limitations. Despite the established efficacy of LBNP at reducing IJV CSA, our data do not indicate SELF LBNP significantly reduced left IJV CSA when compared to the supine condition, suggesting that traditional LBNP may be slightly more effective at reducing IJV CSA when compared to SELF LBNP.

We suggest that the leg press-type exercise required during SELF LBNP may reduce the efficacy of LBNP itself. In function, LBNP reduces interstitial fluid pressure in the lower body, leading to a fluid shift [1]. Contraction of the muscles in the lower body acts to increase the interstitial pressure, which may partially counteract the negative interstitial pressure from LBNP. Furthermore, one study has associated exercise with altered translaminar pressure gradients [17]. These two factors may explain why SELF LBNP was slightly less effective at reducing IJV CSA when compared to traditional LBNP. For example, if local microvascular flow or fluid volume is measured after the LBNP with exercise for a longer time, we may have observed post-exercise hyperemia. Therefore, LBNP and exercise may sequester fluid.

The SELF LBNP prototype has a few noted limitations. The neoprene skirt struggled to maintain an airtight seal on the subject with the smallest waist circumference (68 cm). Our current device would require adjustability features or an additional, smaller neoprene skirt to accommodate smaller waist circumferences. Additionally, shorter subjects could not expand the chamber as much as taller subjects and struggled to reach 25 mmHg. As such, shorter subjects worked harder to produce similar levels of negative pressure when compared to taller subjects. The shortest subject (152 cm) was unable to reach 25 mmHg and their data were excluded from analysis.

This study has limitations that should be acknowledged. First, the sample size was relatively small, comprising only eleven participants (six males and five females), which may limit the generalizability of the findings. Additionally, the study did not consider the menstrual cycle of female participants, which is an oversight as the menstrual cycle can influence physiological responses, such as heart rate and blood pressure, and could potentially impact the results. Future studies should account for this variable to ensure more accurate and comprehensive data. Another limitation is related to the device’s ability to maintain an airtight seal. The neoprene skirt of the SELF LBNP device struggled to maintain a seal on subjects with smaller waist circumferences, potentially affecting the negative pressure achieved. This issue suggests that the device may need further design modifications to accommodate a wider range of body types. Furthermore, the leg press exercise required to operate the SELF LBNP device may have influenced the efficacy of the negative pressure. Muscle contractions can increase interstitial pressure, possibly counteracting the effects of LBNP. This factor could explain why SELF LBNP was slightly less effective at reducing internal jugular vein cross-sectional area compared to traditional LBNP. Lastly, the study was conducted over a short duration, and the long-term effects of SELF LBNP were not evaluated. Future research should include longer trials to better understand the sustained effects and potential benefits of SELF LBNP in simulated microgravity conditions.

With the anticipated growth of space tourism, short-duration missions around Earth’s orbit will become more common. The countermeasure proposed in our study is particularly suitable for these brief exposures to microgravity, offering a practical solution to mitigate physiological changes during such missions.

Future research on the self-generating lower body negative pressure SELF LBNP device should focus on several critical aspects to enhance its applicability and efficacy in space missions. Long-term studies are necessary to evaluate the sustained effects of SELF LBNP on intracranial pressure and internal jugular vein cross-sectional area under extended microgravity conditions. Additionally, refining the device to accommodate a broader range of body types, particularly addressing issues with maintaining an airtight seal, is essential for broader astronaut applicability. Investigating the potential integration of the SELF LBNP device with other countermeasures and its impact on overall astronaut health and performance will provide a holistic understanding of its benefits. These studies could reveal how the device’s unique exercise component influences cardiovascular and musculoskeletal health in microgravity. Ultimately, the successful implementation of SELF LBNP could significantly enhance the quality of life for astronauts on long-duration missions, offering a low-power, space-efficient alternative to traditional methods, thereby contributing to safer and more sustainable deep-space exploration.

A SELF LBNP device is better suited for long-duration spaceflight. The SELF LBNP device has low volume and requires little or no power, which are valuable improvements over traditional LBNP chambers. A SELF LBNP device also has a collapsible design which lends itself to a lower overall mass compared to traditional LBNP chambers. A SELF LBNP device could potentially generate electrical power. Because both the traditional and SELF LBNP conditions reduce IJV CSA to a similar extent, our results suggest dynamic, self-generated LBNP may have a similar effect of reducing ICP when compared to traditional LBNP. Though IJV flow was not explicitly measured, the reduced IJV CSA suggests that SELF may help restore or prevent stagnant venous flow that occurs in some astronauts [14]. These results warrant continued investigation into SELF LBNP. Longer-duration tests in simulated microgravity may provide a more complete characterization of SELF LBNP and help determine whether SELF LBNP may be an effective replacement for traditional LBNP chambers in long-duration spaceflight.

## Figures and Tables

**Table 1:** Wilcoxon signed-rank tests comparing SELF to different conditions. SELF LBNP significantly reduced IJV CSA when compared to supine posture on the right side. However, IJV CSA was not significantly reduced on the left side. Background colors are meant to represent supine configurations.

Configuration #1	Configuration #2	Measurement	Result	*p*-Value
SELF LBNP	Supine	Left	Not different	0.365
SELF LBNP	Supine	Right	Different	0.005
SELF LBNP	Upright	Left	Different	0.032
SELF LBNP	Upright	Right	Different	0.002
SELF LBNP	Traditional LBNP	Left	Not different	0.465
SELF LBNP	Traditional LBNP	Right	Not different	0.577
Traditional LBNP	Supine	Left	Different	0.001
Traditional LBNP	Supine	Right	Different	0.005

## Data Availability

The data presented in this study are available on request from the corresponding author. The data are not publicly available due to participant privacy.
